# The changing profile of SARS-CoV-2 serology in Irish blood donors

**DOI:** 10.1016/j.gloepi.2023.100108

**Published:** 2023-04-21

**Authors:** Dermot Coyne, Dearbhla Butler, Adrienne Meehan, Evan Keogh, Pádraig Williams, Alex Carterson, Tor Hervig, Niamh O'Flaherty, Allison Waters

**Affiliations:** a Irish Blood Transfusion Service, National Blood Centre, James's Street, Dublin D08 NH5R, Ireland; b Centre for Laboratory Medicine and Molecular Pathology, St James's Hospital, James's Street, Dublin D08 NHY1, Ireland; c UCD National Virus Reference Laboratory, University College Dublin, Dublin 4, Ireland; d Abbott Laboratories, 100 Abbott Park Road, Abbott Park, IL 60064, USA; e UCD School of Public Health, Physiotherapy and Sports Science, University College Dublin, Dublin 4, Ireland

**Keywords:** Ireland, SARS-CoV-2, Epidemiology, Blood, Donor, Vaccination, Serology

## Abstract

**Background:**

The present study aimed to investigate the progression of the SARS-CoV-2 pandemic in Ireland over the first three waves of infection.

**Method:**

A selection of blood donor serum samples collected between February 2020 and December 2021 were analysed by various commercially available serological assays for antibodies to SARS-CoV-2 (*n* = 15,066).

**Results:**

An increase in seropositivity was observed between wave 1 (February to September 2020) and wave 2 (November and December 2020) of 2.20% to 3.55%. A large increase in estimated seroprevalence to 11.89% was observed in samples collected in February and March 2021 (wave 3 of infection).The rate of seropositivity varied by age group, with the highest rate observed in the youngest donors (18–29 years) peaking at 18.79% in wave 3. The results of spike antibody (anti-S) testing indicated that 44/1009 (4.36%) of seroreactive donors in wave 3 had a serological profile consistent with vaccination. By November 2021, we detected an overall seropositivity of 97.04%.

**Conclusions:**

The present study provides a comprehensive estimation of the level of circulating SARS-CoV-2 antibodies in Irish blood donors, enabling differentiation between vaccination and natural infection, as well as real-time monitoring of the progression of the COVID-19 pandemic in Ireland. Seroepidemiology has a role in determining reliable estimates of transmission, infection fatality rates and vaccine uptake. The continued screening of blood donors for this purpose has the potential to generate important data to assist with the management of future waves of SARS-CoV-2.

## Highlights

• Report the progression of the SARS-CoV-2 pandemic in Ireland over the first three waves of infection
• By end of 2021, 97% of Irish blood donors were positive for SARs-CoV-2 antibodies
• Vaccination and infection result in different levels of anti-spike production.
• Seroepidemiology has a key role estimating the extent of SARS-CoV-2 pandemic

## Introduction

The World Health Organisation (WHO) issued guidance in May 2020 for population-based age-stratified COVID-19 seroepidemiological investigations recommending suitable populations for studies which included blood donors [1]. Indeed, seroepidemiological assessment of SARS-CoV-2 infection in blood donors assisted with the surveillance and assessment of spread of the COVID-19 pandemic in Ireland [[2], [3], [4]]. We previously reported the detection of SARS-CoV-2 antibodies in Irish blood donors two weeks prior to the first notification of COVID-19 infection in Ireland [5]. Seroprevalence estimates indicate that approximately one third of individuals with detectable SARS-CoV-2 antibodies are asymptomatic [[6], [7], [8]]. Irish COVID-19 surveillance reports indicate that 82% of those with detectable viral RNA were symptomatic at the time of testing over the first year of the pandemic [3]. As a result, and consistent with many infectious disease outbreaks, the full extent of the SARS-CoV-2 pandemic in Ireland is likely under-recorded [7]. Blood donor studies offer a unique opportunity to screen healthy populations for the presence of antibodies to new and emerging infections [[9], [10], [11]].

SARS-CoV-2 infection results in the production of antibodies to many different SARS-CoV-2 antigens, including the viral nucleocaspid (N) and Spike (S) proteins. In contrast, vaccines currently approved in Ireland induce antibody production against the S protein only. Therefore, a combination of anti-N and anti-S antibody screening has been used to distinguish between a serological profile consistent with SARS-CoV-2 infection, and that of immunisation, as anti-N would not develop in response to vaccination alone [1,12]. There are limitations with this approach however, as the lack of a durable anti-N response in some individuals confounding the reporting in many cases [13]. Therefore, the use of serology screening assays as an epidemiological tool for the surveillance of SARS-CoV-2 has had to evolve alongside the pandemic and a comprehensive vaccination programme.

The present study aimed to serologically track the progression of the SARS-CoV-2 pandemic in Ireland over the first three waves of infection and to develop an optimum testing algorithm for differentiating between prior infection and vaccination, thereby adding to the body of epidemiological information employed by public health to respond to an ever-evolving pandemic.

## Materials & methods

### Study design and ethical approval

A selection of anonymised blood donor samples received by the Irish Blood Transfusion Service (IBTS) between February 2020 and December 2021 were analysed for antibodies to SARS-CoV-2 and categorised according to the time period at which they were collected. Due to operational constraints, time points reflecting the major changes in the COVID-19 landscape in Ireland were selected. Samples collected from February to September 2020 represent Wave 1 of the pandemic (*n* = 8509), samples from November to December 2020 represent Wave 2 (*n* = 1014) and samples collected from February to March 2021 represent Wave 3 (*n* = 1009). An additional cohort of samples from October to December 2021 (*n* = 4534) were selected to represent the donor population after the vaccination rollout. This study was approved by the National Office for Research Ethics Committee (www.nrecoffice.ie). Limited demographic information was collected and included age, gender and donation clinic. No clinical information, including symptoms of possible COVID-19 infection, or vaccination status were available.

### Donor SARS-CoV-2 antibody screening

Three antibody screening algorithms were used. The testing strategies were adapted to reflect the increasing availability of commercial assays and the commencement of the national vaccination programme. All testing was carried out as per manufacturer's instructions.

Algorithm 1 Donor serum samples screened during wave 1 and wave 2 were tested with the SARS-CoV-2 IgG assay (Abbott Diagnostics) to detect anti-N**,** SARS-CoV-2 IgG II Quantitative assay (Abbott Diagnostics) targeting anti-S and SARS-CoV-2 Total Antibody assay (Abbott Diagnostics) targeting anti-N and anti-S antibodies. A selection of weakly reactive samples or samples with discordant results (*n* = 40) were referred to the University College Dublin National Virus Reference Laboratory for further analysis as per Butler *et al* [5]**.**

Algorithm 2 Samples from Wave 3, that were reactive with both the Abbott SARS-CoV-2 IgG assay and the Abbott SARS-CoV-2 IgG II Quantitative assay (S+/N+), were classified as indicative of previous infection. In response to evidence in the literature indicating a decline in the sensitivity of the Abbott Architect SARS-CoV-2 IgG assay over time, samples that were anti-S positive and anti-N negative (S+/N-) were further classified according to anti-S quantitative results.

Algorithm 3 Samples from October–December 2021 were screened using the Abbott SARS-CoV-2 IgG II Quantitative Assay, which detects Anti-S. Reactive specimens were referred for testing at Central Pathology Laboratory (CPL), St. James's Hospital using the Roche Elecsys® Anti-SARS-CoV-2 IgG assay which is a highly sensitive assay for the qualitative detection of Anti-N. This work was carried out for the purposes of surveillance work in collaboration with the Health Protection Surveillance Centre.

### Statistical analysis

Statistical analyses were performed using the statistical software package IBM SPSS (Version 27) and MedCalc (www.medcalc.org). Assay performance characteristics were calculated based on the samples from Wave 1 (Algorithm 1) according to the testing algorithm described by Butler *et al* [5]*.* Seroprevalence rates were adjusted for age and sex to reflect the Irish population demographics using the 2016 CENSUS data as per Lewin *et al* [14].

The Chi-Square test and confidence intervals were used to assess associations between donor demographic variables.

## Results

### The changing SARS-CoV-2 seroepidemiological profile during three waves of infection

The SARS-CoV-2 seroprevalence in Irish blood donors after the three waves of infection in Ireland was estimated. Overall, a total of 343/10,533 donor samples were deemed positive for the presence of SARS-CoV-2 antibodies between February 2020 and March 2021. An increase in seropositivity was observed between wave 1 (February to September 2020) and wave 2 (November and December 2020) of 2.20% [95% CI; 1.89 to 2.54] to 3.55% [95% CI; 2.49 to 4.92]. A large increase in estimated seroprevalence to 11.89% [95% CI; 9.89 to 13.89] was observed in samples collected in Wave 3. Wave 3 seroepidemiology was influenced by the vaccination programme which began on the 29th December 2020, starting with healthcare workers and those over 80 years old. Quantitative anti-S testing indicates that 44/1009 (4.36%) of donors in wave 3 had serological profiles consistent with vaccination. SARS-CoV-2 antibody detection rates for donor demographics and time periods are listed in Table 1.

**Table 1:** Donor sample population demographics and SARS-CoV-2 antibody detection for waves 1, 2 and 3.

		Wave 1				Wave 2				Wave 3 (past infection)				Wave 3 (Vaccinated)			
		Total	SARS-CoV-2 Ab detected	Crude Seroprevalence % (95% CI)	Adjusted Seroprevalence % (95% CI)	Total	SARS-CoV-2 Ab detected	Crude Seroprevalence % (95% CI)	Adjusted Seroprevalence % (95% CI)	Total	SARS-CoV-2 Ab detected	Crude Seroprevalence % (95% CI)	Adjusted Seroprevalence % (95% CI)	Total	SARS-CoV-2 Ab detected	Crude Seroprevalence % (95% CI)	Adjusted Seroprevalence % (95% CI)
**Total**		8509	187	2.20% [1.89–2.54]	2.48% [2.16–2.84]	1014	36	3.55% [2.49–4.92]	6.69% [5.21–8.50]	1009	76	7.53% [5.94–9.43]	10.76% [8.87–13.03]	1009	44	4.36% [3.17–5.85]	5.40% [4.11–7.10]
**Gender**																	
	Male	4842	104	2.15% [1.76–2.60]	1.86% [1.50–2.29]	584	19	3.25% [1.96–5.08]	2.79% [1.57–4.45]	580	46	7.93% [5.81–10.58]	6.82% [4.93–9.39]	580	8	1.38% [0.60–2.72]	1.19% [0.49–2.49]
	Female	3667	83	2.26% [1.80–2.81]	2.43% [2.15–3.23]	430	17	3.95% [2.30–6.33]	4.71% [2.84–7.18]	429	30	6.99% [4.72–9.98]	8.32% [5.88–11.62]	429	36	8.39% [5.88–11.62]	10.02% [7.25–13.50]
**Age (years)**																	
	18–29	1133	46	4.06% [2.97–5.41]	5.3% [4.04–6.82]	137	7	5.11% [2.05–10.53]	6.58% [3.00–12.47]	149	18	12.08% [7.16–19.09]	14.23% [8.72–21.54]	149	10	6.71% [3.22–12.34]	8.05% [4.16–14.07]
	30–39	1664	46	2.76% [2.02–3.69]	3.55% [2.70–4.57]	195	7	3.59% [1.44–7.40]	4.65% [2.11–8.76]	197	10	5.08% [2.43–9.34]	6.60% [3.51–11.28]	197	9	4.57% [2.09–8.67]	5.58% [2.79–9.99]
	40–49	2271	39	1.72% [1.22–2.35]	1.50% [1.04–2.09]	264	6	2.27% [0.47–4.07]	5.49% [2.51–10.42]	278	19	6.83% [4.12–10.67]	5.77% [3.29–9.35]	278	8	2.88% [1.24–5.67]	2.52% [1.01–5.19]
	50–59	2241	37	1.65% [1.16–2.28]	1.21% [0.79–1.75]	276	9	3.26% [1.49–6.19]	2.32% [0.80–4.73]	228	17	7.46% [4.34–11.94]	6.58% [3.68–10.85]	228	10	4.39% [2.10–8.07]	3.95% [1.81–7.49]
	≥60	1200	19	1.58% [0.95–2.47]	1.67% [1.02–2.57]	142	7	4.93% [1.98–10.16]	5.31% [2.43–11.10]	157	12	7.64% [3.48–11.80]	9.42% [5.02–16.11]	138	7	5.07% [2.04–10.45]	5.80% [2.50–11.42]
**Province**																	
	Leinster	4955	136	2.74% [2.30–3.25]	2.60% [2.17–3.09]	589	23	3.90% [2.48–5.86]	3.72% [2.34–5.66]	562	52	9.25% [6.85–11.65]	12.33% [9.44–15.92]	485	25	5.15% [3.34–7.61]	5.98% [4.00–8.59]
	Munster	2257	37	1.64% [1.15–2.26]	1.68% [1.19–2.31]	305	12	3.93% [2.03–6.87]	3.61% [1.80–6.45]	323	17	5.26% [3.07–8.43]	4.42% [2.37–7.27]	323	17	5.26% [3.07–8.43]	4.33% [2.37–7.27]
	Connacht	649	5	0.77% [0.25–1.80]	1.23% [0.53–2.43]	34	1	2.94% [0.07–16.39]	8.82% [1.82–25.79]	38	4	10.53% [2.87–26.95]	32.5% [16.32–55.16]	38	0	0.00% [0.00–0.00]	0.00% [0.00–0.00]
	Ulster	648	9	1.39% [0.63–2.63]	1.08% [0.43–2.22]	86	0	0.00% [0.00–0.00]	0.00% [0.00–0.00]	86	3	3.49% [0.72–10.20]	2.33% [0.28–8.40]	86	2	2.33% [0.28–8.40]	1.70% [0.03–6.48]

**Fig. 1:**
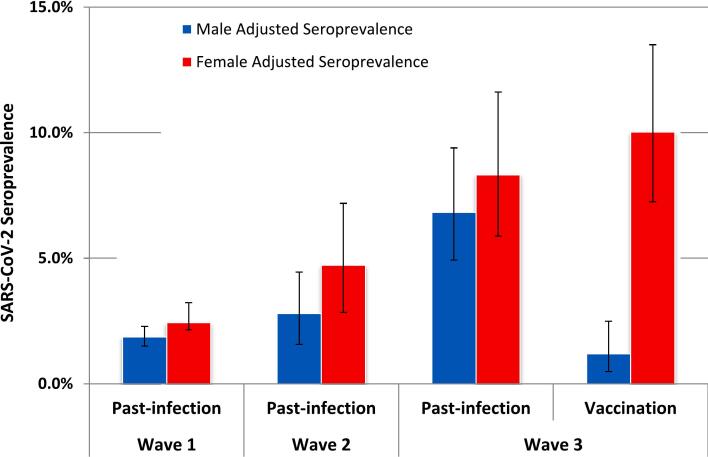
SARS-CoV-2 antibody detection rates in Irish Blood Donations adjusted to National demographic distributions recorded in the 2016 CENSUS. Error bars represent 95% confidence intervals.

There was no difference in the rate of seropositivity between males and females throughout wave 1 and wave 2. However, a higher rate of vaccination was detected in females of 8.39% [95% CI; 5.88–11.62] compared to males, at 1.38% [95% CI; 0.60–2.72] following roll-out of the vaccination programme in wave 3 (Fig. 1). Seroprevalence varied by age group, with the highest rate consistently detected in the youngest age category of 18–29 year olds, peaking at 18.79% [95% CI; 12.52–25.06] in wave 3.

### Evaluation of the SARS-CoV-2 serological screening assays

Performance characteristics of the SARS-CoV-2 assays in the analysis of 8509 anonymised donor specimens from Wave 1 of the pandemic are reported in Table 2. Samples were classified as ‘true’ or ‘false’ positive or negative as per the testing algorithm and based on the result concordance between all assays as per Butler *et al* [5]**.** Samples that were negative by all three screening assays were considered truly negative, those that were positive on at least two out of three screening assays were considered positive. Inconclusive results were referred for additional testing at the National Virus Reference Laboratory using the Fortress Diagnostics Wantai Total antibody assay [5]. Due to the emerging nature of SARS-CoV-2 assays and the absence of a gold standard for SARS-CoV-2 serology, the limitations of the testing algorithms in classifying samples as ‘true’ positive and ‘true’ negative was negated by the inclusion of multiple assays targeting different antibody classes and from different manufacturers.

**Table 2:** SARS-CoV-2 antibody screening assay performance evaluation in healthy blood donors.

Assay		Negative	Grey zone	Positive	Sensitivity % [+/− 95% CI]	Specificity % [+/− 95% CI]	AUC [+/− 95% CI]	PPV % [+/− 95% CI]	NPV % [+/− 95% CI]
Abbott SARS-CoV-2 IgG		8051	341	117					
	aw/o Grey zone	8051	–	117	75.6 [67.0 to 82.9]	99.7 [99.6 to 99.8]	0.877 [0.869 to 0.884]	79.5 [72.0 to 85.4]	99.6 [99.5 to 99.7]
	bGrey zone negative	8392	–	117	55.7 [47.8 to 63.4]	99.7 [99.6 to 99.8]	0.777 [0.768 to 0.786]	79.5 [71.8 to 85.5]	99.1 [99.0 to 99.3]
	cGrey zone positive	8051	–	458	75.6 [67.0 to 82.9]	95.7 [95.2 to 96.1]	0.856 [0.849 to 0.864]	20.3 [18.1 to 22.7]	99.6 [99.5 to 99.7]
Abbott SARS-CoV-2 IgGII Quant		8303	N/A	206	98.8 [95.7 to 99.9]	99.5 [99.3 to 99.6]	0.992 [0.989 to 0.993]	80.1 [74.8 to 84.5]	100.0 [99.9 to 100.0]
Abbott SARS-CoV-2 Total Ab		8315	N/A	194	96.8 [93.1 to 98.8]	99.8 [99.7 to 99.9]	0.983 [0.980 to 0.986]	93.3 [89.0 to 96.0]	99.9 [99.8 to 100.0]
Final ‘True’ Result		8322	N/A	187	–	–	–	–	–

a Grey zone results were excluded from assay performance calculations.

b Grey zone results were classified as negative for assay performance calculations.

c Grey zone results were classified as positive for assay performance calculations.

The SARS-CoV-2 IgG II Quantitative assay demonstrated the best overall performance with a sensitivity and specificity of 98.8% [95% CI; 95.7 to 99.9] and 99.5% [95% CI; 99.3 to 99.6], respectively and a higher area-under-the-curve value (0.992 [95% CI 0.989 to 0.993], *p* < 0.05) compared to the other screening assays, (Table 2). The SARS-CoV-2 Total Ab assay also performed well with a higher positive predictive value than the other screening assays, and a calculated sensitivity and specificity of >96%. However, the interpretation of the Abbott SARS-CoV-2 IgG was challenging owing to the greyzone category. Indeed, reference testing using the Fortress Diagnostics Wantai total antibody assay suggested all 50 ‘greyzone –only’ results (*i.e.* samples without reactivity in any other assay), were falsely reactive. Therefore, for consistency and simplicity ‘greyzone–only’ samples were categorised as antibody negative. We do acknowledge however, that equivocal results such as these may indicate ‘true’ declining antibody levels in the donor sample and further highlight the limitations in the clinical utility of serological analysis using this assay. Inferring all greyzone detections as negative, reduced assay sensitivity to only 55.7% [95% CI; 47.8 to 63.4]. ‘Greyzone-only’ results were excluded from the final calculations of sensitivity and specificity, which were 75.6% [95% CI; 67.0 to 82.9] and 99.7% respectively [95% CI; 99.6 to 99.8].

### Quantitative serological differentiation between COVID-19 vaccination and past infection

**Fig. 2:**
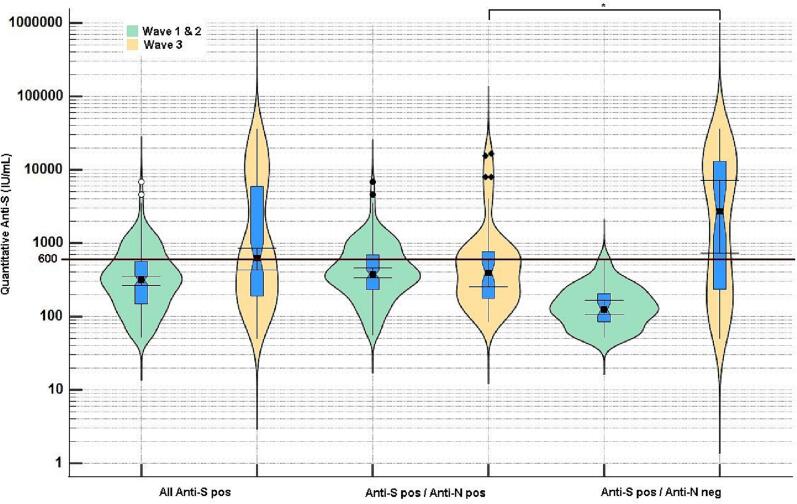
**Quantitative Anti-Spike antibody levels in Irish Blood donors grouped according to time of collection and serological profile.** Marked differences were observed before and after vaccination and quantitative values >600 IU/mL were indicative of vaccination.

In light of the suboptimal sensitivity of the Abbott SARS-CoV-2 IgG assay, an alternative method to discriminate between vaccine-derived immunity and antibody production following infection that would not rely on the sensitivity of the anti-N assay was adopted. The incorporation of the Abbott SARS-CoV-2 IgG II quantitative assay into the testing algorithm enabled quantitative detection of the anti-S antibody concentration. Lower anti-S IgG quantitative values were observed in donor specimens tested prior to the rollout of national COVID-19 vaccination programme. Donor samples that were negative for anti-N, but positive for anti-S, had average quantitative values during wave 1 and 2 of <600 AU/mL (mean: wave 1504.5; wave 2485.2); however, in wave 3, donors with this antibody profile, had higher anti-S quantitative values, with an average value of 5053.1 AU/mL (Fig. 2). The higher SARS-CoV-2 IgG II quantitative result observed in S+/N- donors in samples from wave 3, allowed for the estimation of an anti-S concentration cut off of 600 AU/mL which, in the absence of anti-N reactivity, was suggestive of a vaccine-specific immune response (testing algorithm 2).

### The Impact of vaccination on the seroprevalence of SARS-CoV-2 antibodies

**Fig. 3:**
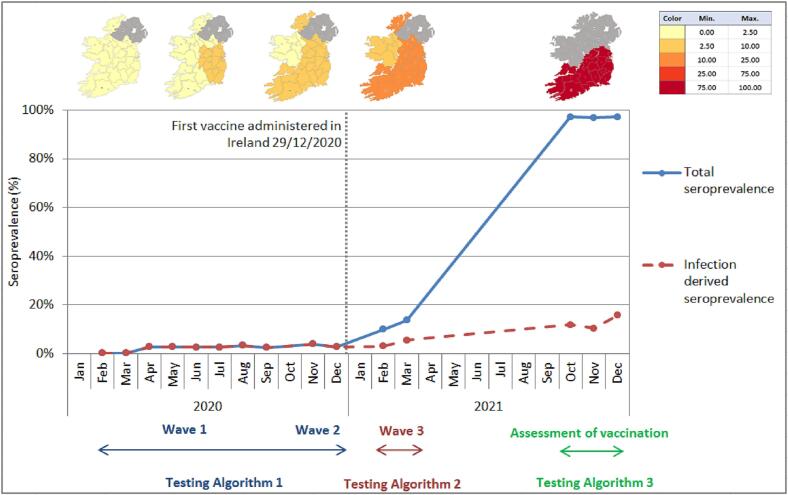
SARS-CoV-2 antibody detection rate in Irish Blood Donors in 2020 and 2021.

By the end of December 2021, the first two doses of COVID-19 vaccinations had been administered to 94.4% of the adult population, and the vaccination booster programme was on-going with over 2 million booster doses administered by the end of December [4]. We report an overall seropositivity of 96.93% [95% CI; 96.17–97.70] in November 2021 compared to 3.41% [95% CI; 1.95–4.85] in November 2020. In contrast the rate of antibody detection due to natural infection during this time was only 12.59% [95% CI; 11.62–13.56] compared to 3.55% [95% CI; 2.49–4.92] one year previously in November and December 2020 (Fig. 3).

## Discussion

Serology has played a key role in both retrospective and real-time modelling of the spread of SARS-CoV-2 infection and is useful in tracking asymptomatic transmission in ‘healthy’ individuals, such as healthcare workers and blood donors [1,4,11,15]. The results of the present study are consistent with previous findings on the performance of SARS-CoV-2 antibody diagnostic assays [16]. However, the majority of performance evaluation studies of SARS-CoV-2 serology assays have typically assessed sensitivity using samples from SARS-CoV-2 PCR positive patients at known time points after the onset of symptoms. We describe a healthy blood donor population, in which the Abbott SARS-CoV-2 IgG assay yielded inconsistent results. Donor results that were scattered throughout the entire “greyzone” range of the Abbott SARS-CoV-2 IgG assay were classified as either serologically positive or negative, following repeat testing using another assay. It is possible that limiting the greyzone range may improve assay specificity but conversely may impact the identification of true positive samples in the clinical setting. The sensitivity of the Abbott SARS-CoV-2 IgG assay has been reported to reduce to 71% at >81 days post diagnosis [17]. In addition, 10–30% of healthcare workers in the United Kingdom with mild SARS-CoV-2 infection have had negative results with this assay, yielding a reported sensitivity of 79.3% in mildly symptomatic individuals [18], and we conclude is not sufficiently sensitive as screening tool for asymptomatic infections in blood donors.

The “Adapting UNITY study protocols to COVID-19 vaccine” recommends using two highly sensitive serological assays to accurately determine the proportion of the population that have vaccine-induced antibodies to SARS-CoV-2 and those with serological profiles consistent with prior infection [19]. Our results provide evidence that the Abbott SARS-CoV-2 IgG II quantitative assay is highly sensitive and specific, and is a suitable assay for this application. A recent comprehensive evaluation of this assay demonstrated its potential capacity for monitoring the immune response to natural infection and its ability to detect antibodies in patients with two SARS-CoV-2 new variants of concern [20]. The detection of SARS-CoV-2 anti-spike antibodies using the Abbott SARS-CoV-2 IgG II assay correlates well with the presence of functional neutralizing antibodies and is highly sensitive for the quantitative detection of vaccine specific antibody responses [21]**.** Therefore, quantitative anti-S screening may also have a role in evaluating the efficacy of the various SARS-CoV-2 vaccines. The reason for the notable difference in the detection of anti-S between men and women remains unclear and may relate to the preponderance of females healthcare workers in Ireland. In addition, blood donation eligibility criteria has changed throughout the pandemic and many healthcare workers working in high-risk wards, were initially deferred from donation or advised not to attend.

Throughout 2021, further doses of COVID-19 vaccines were administered at various rates in different age groups, concurrent with the emergence of viral variants of concern and cases of reinfection. The WHO published enhanced guidelines to assist in the differentiation of vaccine-derived immunity *versus* infection-derived immunity and recommended the inclusion of a sensitive anti-N screening assay as a more robust method for the differentiation of antibody responses [1]. The present study introduced the Roche Elecsys Anti-SARS-CoV-2 immunoassay in response to this recommendation, which detects total antibodies (including IgG) against the SARS-CoV-2 N antigen and has reported better performance compared to the Abbott SARS-CoV-2 IgG assay in a number of studies [22,23]. The higher sensitivity in detecting antibodies to the N antigen could be in part because the Roche assay detects multiple classes of antibodies compared to the Abbott assay, which exclusively detects IgG antibodies. In addition to this the Roche assay has demonstrated a superior ability in maintained detection of antibodies up to at least 7 months compared to the Abbott assay, making it more suitable for identifying infection-derived immunity [24].

High concentrations of circulating anti-S, detected in our donors after the initiation of the vaccination programme, were used as a secondary factor to distinguish between COVID-19 vaccination as opposed to past infection, prior to the publication of the revised WHO testing algorithm and the implementation of the more sensitive Roche Elecsys anti-N assay. The finding that vaccines induce higher quantitative antibody levels compared to natural infection has been reported in numerous studies to date [25,26]. One item for consideration is that of hybrid immunity, which is defined as the immune protection in individuals who have had one or more doses of a COVID-19 vaccine, and experienced at least one SARS-CoV-2 infection before or after the initiation of vaccination [27]. A single booster dose given to previously infected patients raised an antibody response significantly higher than two doses given to naïve individuals. In addition heterologous vaccination generated a robust persistent antibody response at high levels, steady up to three months after administration [27]. It was not possible to distinguish hybrid immunity from those that were previously vaccinated only in the anonymised donor sample population, and therefore some hybrid immunity may have been misclassified as vaccine-derived immunity if the anti-N assay failed to detect their anti-N antibody due to poor assay sensitivity.

The use of serology for diagnosis and surveillance of respiratory viruses has limitations and is not routinely performed for other viral infections. In addition, blood donors are likely to differ from the general population in terms of sociodemographic, behavioural and health-related factors. Indeed, the exposure to various strains of the seasonal influenza virus, reinfection, antigenic drift and vaccination leads to complex individual antibody profiles [28,29]. However, Pandemic Preparedness and Response Planning includes seroepidemiology as a key surveillance initiative that is required at the start of any pandemic as it has a role in determining reliable estimates of transmission, tracing, infection fatality rates and impact of emerging pathogens [10,19,30]. Serological studies can inform age-related morbidity and mortality modelling, supporting estimates for the provision of vaccines and antiviral medications [10,19,30]. Due to the developing nature of SARS-CoV-2 serology assays and the absence of a gold standard for SARS-CoV-2 serology, the testing algorithms used in this study to classify samples as truly positive or truly negative was limited by the sensitivity and specificity of the assays used. We attempted to account for this by using multiple assays targeting various antibody classes and viral targets. However it must be noted that classifying S + N- as indicative of vaccination and non-exposure to the SARS-Cov-2 Virus is not a perfect method as it may exclude previously infected individuals where anti-N has declined rapidly compared to anti-S, and is potentially no longer detected by a screening assay [[31], [32], [33]].

The present study clearly demonstrated the remarkable impact that vaccination had in increasing the total seropositivity by November 2021 and is consistent with the vaccine uptake estimated for the general Irish population. Overall, a total of 93.4% Irish adults were estimated to be fully vaccinated at the end of November 2021, which is one of the highest vaccine uptake rates in the European Union [3]. The continued screening of blood donors has the potential to generate important data, assisting with the management of future waves of infection and supporting the development of appropriate national vaccination strategies.

## Financial support

This work was supported by the Irish Blood Transfusion Service internal Research and Development Fund, and by 10.13039/100014386Abbott Diagnostics, United States. Abbott diagnostics provided reagents for testing; however, has had no involvement in study design; sample collection, analysis and interpretation of data; writing of the report; and in the decision to submit the article for publication.

## CRediT authorship contribution statement

**Dermot Coyne:** Methodology, Data curation, Supervision, Funding acquisition. **Dearbhla Butler:** Methodology, Data curation, Formal analysis, Writing – original draft. **Adrienne Meehan:** Methodology, Data curation. **Evan Keogh:** Methodology, Data curation. **Pádraig Williams:** Resources, Project administration. **Alex Carterson:** Methodology, Funding acquisition. **Tor Hervig:** Funding acquisition. **Niamh O'Flaherty:** Conceptualization, Methodology, Writing – review & editing, Project administration, Supervision. **Allison Waters:** Data curation, Writing – original draft, Project administration, Formal analysis.

## Declaration of Competing Interest

The authors declare the following financial interests/personal relationships which may be considered as potential competing interests:

This work was supported by the Irish Blood Transfusion Service internal research and development funding, and by Abbott Diagnostics. Abbott diagnostics provided reagents for testing; however, has had no involvement in study design; sample collection, analysis and interpretation of data; writing of the report; and in the decision to submit the article for publication.
